# Editorial: Unconventional Animal Models in Infectious Disease Research

**DOI:** 10.3389/fcimb.2021.762581

**Published:** 2021-10-26

**Authors:** Layla Kamareddine

**Affiliations:** ^1^ Department of Biomedical Sciences, College of Health Sciences, QU Health, Qatar University, Doha, Qatar; ^2^ Biomedical Research Center, Qatar University, Doha, Qatar; ^3^ Biomedical and Pharmaceutical Research Unit, QU Health, Qatar University, Doha, Qatar

**Keywords:** unconventional animal models, host-pathogen interactions, infectious disease research, treatment targets, *in vivo* studies

  Beyond an *in vitro* setting, the use of a biological system is considered imperious to unravel the enigma of host-pathogen interactions, particularly those eventuating in an infectious disease scenery. In the past two-decades, the use of animal models, especially the unconventional ones, in studying infectious disease occurrence and progression has stemmed out. This rise in the use of animal models in research was greatly reinforced by the furtherance advancements in the field of genetics that has opened up for feasible genetic manipulation of both the host and the pathogen as needed throughout the course of conducted studies. Within this frame of reference, our launched topic envisioned to bring in research work that have used a broad spectrum of unconventional animal models to introduce pioneering findings in host-pathogen interaction studies. These findings will not only shed more light on our understanding of host-pathogen interfaces, but also set the foundation for innovative therapeutic regimens to control infectious diseases.

In the first contributed article to our Research Topic (Li et al.), the authors described the characteristics of the duck enteritis virus (DEV) ICP22 protein and explored its role in DEV replication. Their findings present ICP22 as a non-essential immediate early protein chiefly located in the nucleus of infected duck embryo fibroblasts cells and reveal that ICP22 encloses a classical nuclear localization signal at 305-314 AA, with a need of residue 309 for ICP22 nuclear localization. Moreover, their verdicts evidently highlight the role of the US1 gene encoding ICP22 in DEV replication. One potential benefit of this finding could be implicated in combating Duck plague infectious disease caused by DEV.

Within the poultry viral infectious disease contexture as well, and as part of the ongoing search for novel anti-viral control strategies, the second contributed article to our Research Topic (Hu et al.) present autophagy as a potential therapeutic target for Duck tembusu virus (DTMUV) infection. In this study conducted on ducks as the animal model of choice, and as a follow up on their previous *in vitro* work divulging that autophagy promotes DTMUV replication (Hu et al., 2020), the authors show that autophagy is triggered in a DTMUV infection setting and that autophagy inhibitors impedes DTMUV replication and attenuates DTMUV-caused pathological manifestations possibly through a host innate immune response-dependent manner.

Owing to the importance of using *Drosophila melanogaster* in infectious disease research and to our recently achieved ground-breaking findings in host-pathogen interaction studies using the fruit fly model organism (Kamareddine et al., 2018a; Kamareddine et al., 2018b; Jugder et al., 2021), the third contribution to this Research Topic was a review put up by our group to distinctly highlight the importance and advantages of *Drosophila* as a model organism of choice in host-pathogen interactions studies (Younes et al.). Communally, this review, along with others including another review from our group on a similar subject matter (Kamareddine et al., 2020) aim to provide comprehensive insight into the use of animal models in disease research.

Besides *Drosophila*, zebrafish also gained great popularity in infectious disease research as a prime model organism. Several studies including that of Johansen and Kremer submitted to our Research Topic, for example, employed the use of zebrafish embryos to unravel the pathogenesis of human diseases. In their study herein, the authors investigated the disease pathogenesis entangled in *Mycobacterium fortuitum* infection and revealed a role of the cystic fibrosis transmembrane conductance regulator (CFTR) in host immune defense against this pulmonary and extra-pulmonary infection-causing bacteria.

Within the same notion of implementing animal models to decipher key players involved in immune defense mechanisms against invading pathogens, Zhang et al.’s study in this Research Topic exploited the *Anopheles gambiae* mosquito model, along with genetic and biochemical assays, to underline the role of the clip domain serine protease CLIPB10 in the regulatory network controlling the melanization immune response against the malaria parasites. The findings of this study acquaints previous similar work from this research group on the *Anopheles gambiae* model organism, mainly those directed towards unraveling the molecular mechanisms of different innate immune arms in the malaria vector against invading pathogens (Yassine et al., 2012; Yassine et al., 2014; Kamareddine et al., 2016; Zhang et al., 2016; El Moussawi et al., 2019).

Lately, the greater wax moth, *Galleria mellonella*, has been also introduced as a new-fangled model for host-pathogen interaction studies (Bismuth et al., 2019). In their contributed work to this Research Topic, Ménard et al. present larval staged *Galleria mellonella* as a fast and easy system to use for investigating the role of small regulatory RNAs (sRNAs) in *staphylococcus aureus* virulence, opening up for similar future studies on other human bacterial pathogens using this model organism. Likewise, Revtovich et al.’s contribution to this Research Topic represent the first developed and characterized “high-throughput *C. elegans–E. faecium* infection model” used for identifying a spectrum of virulence genes that take part in the pathogenesis of the bacterial infection. Such an established platform could be eventually built on to introduce novel treatment approaches for *E. faecium* infection, fundamentally those emerging in hospital settings.

Although our Research Topic was particularly directed towards the use of unconventional animal models in infectious disease research, mice as traditional model organisms have always served as a basic platform for *in vivo* studies. As such, we welcomed the contribution of Ji et al. to this Research Topic whose work relied on the use of a neonatal mouse necrotizing enterocolitis (NEC) model to study the effect of exogenous Autoinducer-2 (AI-2) on intestinal dysbiosis and inflammation and therefore clinch a role of AI-2 in partially rescuing flora disruption and decreasing the inflammatory response in the mouse NEC model.

Owing to the outstretched success we achieved in this volume of our Research Topic, we have now launched volume II under the same topic title in which we welcome more submissions and aim to initiate a useful foundation for interested scientists in this subject matter.

## Author Contributions

The author confirms being the sole contributor to this editorial and has approved it for publication.

## Conflict of Interest

The author declares that the research was conducted in the absence of any commercial or financial relationships that could be construed as a potential conflict of interest.

## Publisher’s Note

All claims expressed in this article are solely those of the authors and do not necessarily represent those of their affiliated organizations, or those of the publisher, the editors and the reviewers. Any product that may be evaluated in this article, or claim that may be made by its manufacturer, is not guaranteed or endorsed by the publisher.
